# Evolutionary algorithm for the optimization of meal intake and insulin administration in patients with type 2 diabetes

**DOI:** 10.3389/fphys.2023.1149698

**Published:** 2023-04-06

**Authors:** Eva Gonzalez-Flo, Elaheh Kheirabadi, Carlos Rodriguez-Caso, Javier Macía

**Affiliations:** ^1^ Synthetic Biology for Biomedical Applications, Department of Medicine and Living Sciences, Universitat Pompeu Fabra, Barcelona, Spain; ^2^ Department of Molecular Biology and Biochemistry, Faculty of Sciences, Andalucía Tech, University of Malaga, Malaga, Spain; ^3^ IBIMA (Biomedical Research Institute of Malaga), Malaga, Spain

**Keywords:** diabetes type 2 (T2D), evolutionary algorithm (EA), computational modeling, AI in diabetes, glyceamia regulation

## Abstract

The optimal management of type 2 diabetes (T2DM) is complex and involves an appropriate combination of diet, exercise, and different pharmacological treatments. Artificial intelligence-based tools have been shown to be very useful for the diagnosis and treatment of diverse pathologies, including diabetes. In the present study, we present a proof of concept of the potential of an evolutionary algorithm to optimize the meal size, timing and insulin dose for the control of glycemia. We found that an appropriate distribution of food intake throughout the day permits a reduction in the insulin dose required to maintain glycemia within the range recommended by the American Diabetes Association for patients with T2DM of a range of severities. Furthermore, the effects of restrictions to both the timing and amount of food ingested were assessed, and we found that an increase in the amount of insulin was required to control glycemia as dietary intake became more restricted. In the near future, the use of these computational tools should permit patients with T2DM to optimize their personal meal schedule and insulin dose, according to the severity of their diabetes.

## 1 Introduction

Type 2 diabetes mellitus (T2DM) is a metabolic disorder characterized by hyperglycemia secondary to insulin resistance and relative insulin deficiency (Gardner, 2011). An impairment in the ability of cells to respond appropriately to insulin exists in muscle, liver, and adipose tissue (Cantley and Ashcroft, 2015) (Clark et al., 2001) (Jeong et al., 2010). The most common consequences of this defect, if left untreated, are i) blindness, ii) renal failure, iii) myocardial infarction, iv) stroke, and v) abnormal blood flow in the extremities, which can necessitate amputation and is associated with premature death (Fonseca, 2009) (Papatheodorou et al., 2018).

T2DM is a disorder that typically manifests in adults; is the most common form of diabetes, accounting for 80%–90% of all cases; and is increasing in prevalence worldwide. Indeed, the global prevalence of T2DM is projected to increase to 7.079 individuals per 100.000 by 2030. In addition, there is a concerning rise in prevalence in lower income countries. Thus, urgent public health and clinical preventive measures are required (Safiri et al., 2022) (Wareham and Herman, 2016).

The principal causes of T2DM are obesity, a sedentary lifestyle, and genetic factors. In particular, obesity is considered to be the principal cause of T2DM in genetically predisposed individuals (Ismail et al., 2021) (Wu et al., 2014). In the early stages of the disease, it can be controlled through an increase in exercise and changes to the diet (Colberg et al., 2010) (Kirwan et al., 2017) (Nelson et al., 2002). However, during the later stages, medications such as metformin (Rojas and Gomes, 2013) and ultimately insulin (Swinnen et al., 2009) are required. Insulin administration is recommended for patients with glycated hemoglobin levels >9% and those whose diabetes cannot be controlled using oral glycemic therapy (Marín-Peñalver et al., 2016) (Thrasher, 2017).

Good glycemic control is defined by the American Diabetes Association (ADA) using blood glucose concentrations between 80 and 130 mg/dL before meals and <180 mg/dL 2 h following meals (Care and Suppl, 2022). The optimal type of insulin therapy varies according to the characteristics of each patient: to achieve the recommended glucose concentrations, basal insulin alone, prandial insulin alone, or a combination of the two may be required.

Specifically, in this study the use of insulin pumps for insulin supply is considered. Despite only around0.5%–1.0% of Type 2 patients use insulin pumps, continuous pump administration has been found to result in improved glycemic control, making this a good option for improving life expectancy overall (Freckmann et al., 2021). For this reason, the study of new approaches to optimize the use of this type of device in type 2 patients is of interest.

Although insulin is a highly effective therapy for diabetes, the use of exogenous insulin is been associated with a number of side effects, including weight gain, a worsening of diabetic retinopathy, changes in the refractive properties of the lens, dizziness, and difficulty breathing (Lebovitz, 2011) (Holman et al., 2009). The most common side effect is hypoglycemia, owing to a mismatch between food intake and the insulin dose being administered. Thus, to appropriately control diabetes, it is necessary to optimize the diet of each patient in terms of the quantity and type of food, the pattern of food consumption during the day, and exogenous insulin administration. Many methods can be used to determine the amount of food that should be consumed and the amount of insulin required for glycemic control, but these must be adapted to the specific characteristics of the patient using a trial and error method.

In the present study, we present a proof of concept of how an optimization process based on an evolutionary algorithm (EA) allows to determine the optimal pattern of dietary intake and the optimal insulin doses for patients with T2DM administering insulin therapy treatment. We optimized these parameters according to the characteristics of each patient, with three specific objectives: i) to prevent episodes of hypoglycemia, ii) to minimize the severity of hyperglycemia, and iii) to minimize the insulin requirement. It is worth noting that despite the extensive study of the use of AI techniques in diabetes (Li et al., 2021) (Sapna et al., 2012) (Alharbi and Alghahtani, 2019), only a few previous studies have explored the use of EAs to optimize the diet of patients with diabetes (Kane et al., 2016).

To evaluate the use of EAs for the optimization of the control of T2DM, a mathematical model was used to describe the physiology of a virtual patient with T2DM. The use of mathematical models in preclinical trials is becoming increasingly widespread because they accelerate the development of new therapies. For example, the US Food and Drug Administration accepts a DM1 simulator as a substitute for preclinical trials for certain insulin therapies, including a closed-loop algorithm for use with an artificial pancreas (Dalla Man et al., 2014).

The model used in the present study was an adaptation of the model developed by Visentin et al (2020), involving a basal insulin infusion and a prandial insulin bolus. The parameters included permit individuals with differing severities of T2DM to be modeled.

We developed the EA with the objective of determining the optimal daily food intake pattern and optimal insulin therapy (such as prandial insulin in combination with basal insulin administration), and minimizing the total insulin dose required.

## 2 Materials and methods

### 2.1 Mathematical model describing the physiology of the participants

To evaluate the effects of differing intake patterns on glycemic control *in silico*, we implemented a mathematical model that described the physiology of patients with T2DM, based on the model developed by Visentin et al (2020). The model described glucose transit through the gastrointestinal tract, the effects of insulin on glucose utilization and production, and the control of insulin secretion by glucose.

The model constructed was a combination of submodels that describe various processes (Supplementary Figure S1). Specifically, the submodels included within the model constructed were as follows: 1) the glucose subsystem, 2) intestinal glucose absorption, 3) renal glucose excretion, 4) endogenous glucose production, 5) glucose use, and 6) the insulin subsystem. For simplicity, the C-peptide secretion system was not considered because does not have backward regulations affecting the other systems involved (see Figure 2 in Visentin et al 2020). The Supplementary material contains a detailed description of all the subsystems considered and the parameters used in the simulations.

In the present study, a subcutaneous insulin subsystem was added to this model to simulate subcutaneous insulin infusion and prandial administration, according to the model developed by Schiavon et al. (2020) and as described inSupplementary Equation S9.

It should be noted that this model provides a description of patients with T2DM and a range of levels of insulin production and insulin resistance, which depend on their i) levels of peripheral and hepatic insulin sensitivity, ii) level of *β*-cell responsivity, and iii) basal circulating insulin and glucose concentrations.

Three individuals with differing severity of T2DM were studied: T2DMA, corresponding to a person with prediabetes, and T2DMB and T2DMC, corresponding to patients with intermediate and advanced stages of diabetes, respectively. The parameters used to describe these individuals were as follows: i) basal insulin (Ib), ii) basal glucose (Gb), iii) insulin-dependent glucose utilization (Vmax), iv) pancreatic responsivity to the glucose rate of change K), v) pancreatic insulin secretion *β*), and vi) the response of the liver to insulin with respect to endogenous glucose production (kp3). Table 1 shows the parameters of each of these individuals. The rest of parameters are shown in Supplementary Table S1.

**TABLE 1:** Characteristics of the individuals analyzed.

Parameter	T2DMA	T2DMB	T2DMC
Ib	57.9	59.3	60.3
Gb	120.8	146.1	161.8
Vmax	0.042	0.039	0.032
K	397	270.1	150.2
β	27.3	20	12.1
Kp3	0.007	0.006	0.005
Body mass (kg)	74	74	74

### 2.2 Evolutionary algorithm

To achieve the objective of identifying a dietary intake pattern that optimizes glucose control and minimizes the insulin dose, we implemented an EA that includes the following:

a. The total dietary intake throughout the day, resulting in the liberation of 195 g of glucose by digestion.
b. A set of N different intake patterns $\left\{P_{1},P_{2},\ldots P_{N}\right\}$ , with N = 10.000.
c. A maximum of five meals for each pattern. Each $P_{j}$ pattern was defined as a set of values $\left(Q_{i}^{j},T_{i}^{j},D_{i}^{j}\right)$ , i.e., $P_{j}=\left\{\left(Q_{1}^{j},T_{1}^{j},D_{1}^{j}\right),\left(Q_{2}^{j},T_{2}^{j},D_{2}^{j}\right),\ldots ,\left(Q_{5}^{j},T_{5}^{j},D_{5}^{j}\right)\right\}$ ( j≤N ), where $Q_{i}^{j}$ is the amount of glucose ingested, and $T_{i}^{j}$ is the time of meal consumption. Finally, each meal was combined with one dose of prandial insulin, $D_{i}^{j}$ , i≤5 , and $\sum_{i=1}^{5}Q_{i}^{j}=195$ g. Finally, the meal pattern $P_{j}$ was combined with a basal infusion of insulin ${I_{B}}_{j}$ from a pump.
d. Combinations of diet, prandial insulin, and basal insulin, i.e., $\Psi_{j}=\left(P_{j},{I_{B}}_{j}\right)$ , each associated with a fitness function $f_{j}$ that quantified the adequacy of glycemic regulation. This fitness function was defined as follows:

$$f_{j}=\mu_{1}\cdot f_{L}+\mu_{2}\cdot f_{H}+\mu_{3}\cdot \;{I_{B}}_{j}+\left(1-\mu_{1}-\mu_{2}-\mu_{3}\right)\cdot \sum_{i=1}^{5}D_{i}^{j}$$ (1)

where

$$f_{L}=\left\{\begin{array}{c} 0\;if\;G_{\min}\in \left(80,100\right)\\ \left|G_{\min}-G_{0}^{t}\right|\;otherwise \end{array}\right.$$

and

$$f_{H}=\left\{\begin{array}{c} 0\;if\;G_{\max}<G_{1}^{t}\\ \left|G_{\min}-G_{0}^{t}\right|\;otherwise \end{array}\right.$$

Here, $G_{\min}$ and $G_{\max}$ are the minimum and maximum plasma glucose concentrations during the day, $G_{0}^{t}$ is the target minimum value for plasma glucose, and $G_{1}^{t}$ is the target 2 h postprandial plasma glucose concentration. Simulations were performed with $G_{0}^{t}=90$ mg/dL and $G_{0}^{t}=170$ mg/dL.

The first term $f_{L}$ represents the difference between the real minimum plasma glucose concentration and the optimum concentration Gt , the second term is the maximum change in plasma glucose induced by each meal, the third term is the basal insulin dose, and the final term is the total prandial insulin dose administered during a day. The parameters $\mu_{1}$ , $\mu_{2}$ , and $\mu_{3}$ correspond to the weightings of the contribution of each of these terms to the $f_{j}$ function. In the simulations performed, $\mu_{1}=0.35$ , $\mu_{2}=0.35$ , and $\mu_{3}$ = 0.15 were used, to prioritize the avoidance of hypoglycemic episodes over insulin dose.

The objective of the EA was to minimize this fitness function. To this end, it included the following steps:

Step 1: The N elements $\Psi_{j}$ were initially randomly determined (the size of each meal $Q_{i}^{j}$ , the time of meal ingestion $T_{i}^{j}$ , the dose of prandial insulin $D_{i}^{j}$ , and the level of the basal infusion ${I_{B}}_{j}$ ).

Step 2: For each of the individuals described in Table 1, all the combinations of $\Psi_{j}$ were applied, and the physiological responses were computationally simulated.

Step 3: The fitness function $f_{j}$ was calculated for each $\Psi_{j}$ .

Step 4: The $\Psi_{j}$ combinations were sorted from the lowest to highest values of $f_{j}.$ If two or more combinations had the same values of $f_{j}$ , they were sorted from the lowest to highest dose of total insulin administered, i.e., the sum of the prandial and basal insulin doses.

Step 5: 25% of the $\Psi_{j}$ with the lowest $f_{j}$ values were selected, and the rest were discarded.

Step 6: A new set of $\Psi_{j}$ was generated. For this purpose, the 25% selected in the previous step were retained, and new combinations were created until N elements were achieved. These new combinations were the result of the duplication $\Psi_{j}$ randomly chosen with probability $\eta_{j}$ from those selected in step 5, along with random mutations. The probability of selection was calculated as follows:

$$\eta_{j}=\frac{\frac{1}{f_{j}}}{\sum_{k=1}^{N}\frac{1}{f_{k}}}$$ (2)

Once a combination $\Psi_{j}$ was selected, an element of the set { $Q_{i}^{s}$ , $T_{i}^{s}$ , $D_{i}^{s}$ , ${I_{B}}_{s}$ } was randomly selected to be mutated with equal probability. The mutation consisted of a random variation $\varepsilon \in \left[-\rho ,+\rho \right]$ of the selected element, where ρ is the maximum amplitude of mutation. In all the simulations performed, ρ=0.1 was used. Where the element to be mutated was the size of the meal, i.e., $Q_{i}^{s}$ , the change in this parameter was transferred to another meal size $Q_{k}^{s}$ that was randomly chosen, to maintain the same total food intake during a day. Table 2 summarizes the rules for the mutation.

**TABLE 2:** Rules for mutation.

Element	Value before mutation	Value after mutation
Size of meal	$Q_{i}^{s}$	$Q_{i}^{s}\cdot \left(1+\varepsilon \right)$
	$Q_{k}^{s}$	$Q_{k}^{s}\cdot \left(1-\varepsilon \right)$
Time of meal	$T_{i}^{s}$	$T_{i}^{s}\cdot \left(1+\varepsilon \right)$
Prandial insulin dose	$D_{i}^{s}$	$D_{i}^{s}\cdot \left(1+\varepsilon \right)$
Basal insulin infusion	${I_{B}}_{s}$	${I_{B}}_{s}\cdot \left(1+\varepsilon \right)$

Step 6 was repeated until a group of N combinations was compiled, which was used to configure the next-generation of the evolutionary process. Subsequently, the algorithm returned to step 2 and was repeated until the maximum number of generations was reached.

It should be noted that this EA did not include crossover between $\Psi_{j}$ combinations because it is necessary to ensure that the total food intake remained constant.

Supplementary Figure S2 summarizes the different steps involved in this evolutionary process.

## 3 Results

### 3.1 Characteristics of the individuals studied

**FIGURE 1:**
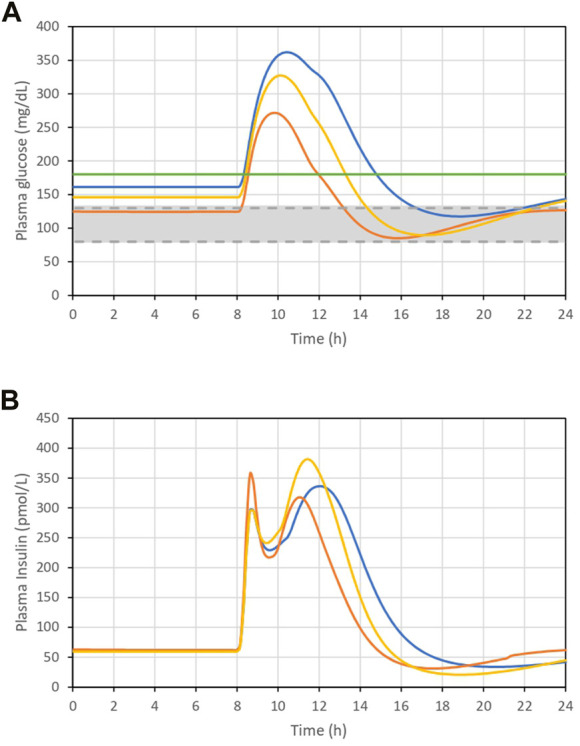
**(A)**. Plasma glucose concentration curve following the ingestion of 115 g glucose. **(B)**. Plasma insulin concentration curve following the ingestion of 115 g glucose. T2DMA (orange line), T2DMB (yellow line), and T2DMC (blue line): individuals with increasing severity of type 2 diabetes. Green solid line is the limit of 180 mg/dL. Grey area represents the interval between 80 and 130 mg/dL.

To study the utility of EAs for the optimization of dietary intake and insulin dose necessary to facilitate glycemic control, three individuals with differing severities of T2DM were considered: T2DMA, T2DMB, and T2DMC, plus a healthy individual as a reference. The parameters defining the characteristics of each individual are presented in Table 1. The remaining model parameters were identical for T2DMA, T2DMB, and T2DMC (Supplementary Table S1). Figure 1A shows the glucose curves obtained for individuals following the consumption of 115 g of glucose in the absence of exogenous insulin administration. This figure shows in gray the zone considered to represent normoglycemia, according to the ADA criteria, and prior to the glucose load, the plasma glucose levels of the participants should have been within this zone. According to the same criteria, 2 h following a meal, the glucose curves should have been in the yellow zone, which corresponded to plasma glucose concentrations <180 mg/dL. The simulation results presented in Figure 1A show differing degrees of deviation from the ADA criteria, corresponding to differing severities of diabetes. It can be seen how the individual T2DMA showed poor glucoregulation following food intake but acceptable basal plasma glucose concentrations (within the gray area), corresponding to prediabetes. The individuals T2DMB and T2DMC showed poorer regulation of both basal and postprandial glucose concentrations.

Figure 1B shows the plasma insulin concentrations of these individuals. Their plasma insulin concentrations were found to be higher than in healthy individuals as a result of insulin resistance. However, these concentrations decreased as glucose regulation worsened, which implies that in addition to insulin resistance, there was a reduction in the ability of the individuals to secrete insulin, corresponding to a worsening of diabetes.

### 3.2 Optimization of food consumption and insulin administration patterns

The EA described in the Materials and methods section was used to determine the food intake pattern that would minimize the dose of insulin, both basal and prandial, necessary to achieve good glucoregulation, according to the ADA criteria.

In the first set of simulations, the EA was permitted to introduce random mutations with respect to both the insulin dose and the timing and size of each meal. Specifically, in each simulation, the consumption of a meal was regarded as taking 20 min (Supplementary Equation S4), basal insulin was assumed to be supplied using an insulin pump at a constant infusion rate during the day, and prandial insulin was assumed to be injected 15 min before food intake (Supplementary Equation S9). In all the simulations performed, the total amount of glucose consumed during each day was 195 g, distributed across up to five meals. These meals were stipulated to be consumed within a maximum of a 14 h period. The simulations were conducted over 600 generations, after which the results had clearly stabilized.

**FIGURE 2:**
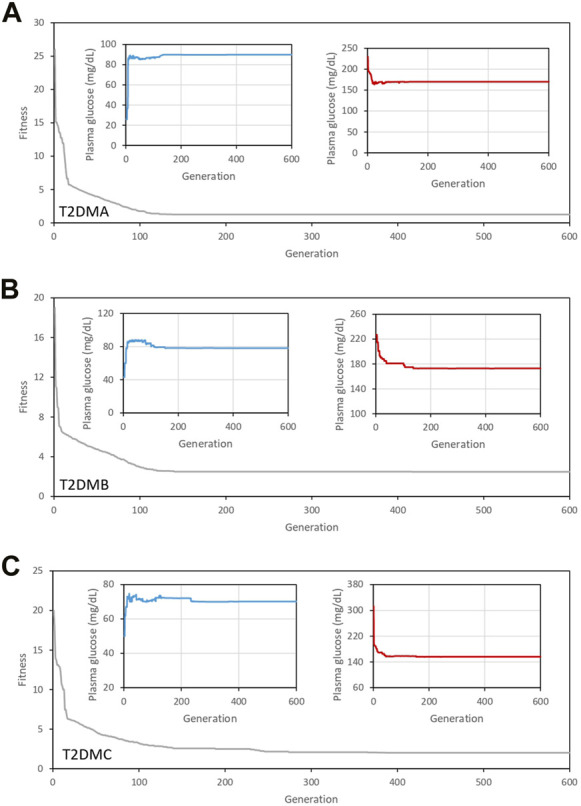
Evolution of the fitness function over 600 generations for the three individuals studied (gray line). **(A)** T2DMA, **(B)** T2DMB, and **(C)** T2DMC. All the simulations were performed using the parameters $\mu_{1}=0.35$ ; $\mu_{2}=0.35$ ,; $\mu_{1}=0.15$ for the calculation of the fitness function. Inset figures represent the minimum (blue line) and maximum (red line) plasma glucose concentrations 2 h following meal consumption. The gray area represents the optimal basal glucose concentrations for patients with diabetes, according to the American Diabetes Association criteria.

Figure 2 shows the results of the first set of simulations. Figures 2A–C show the evolution of the fitness function over successive generations for the three individuals. As shown in the figures, after 600 generations, the fitness function was stable. All the simulations were performed using the parameters $\mu_{1}=0.35$ , $\mu_{2}=0.35$ , and $\mu_{1}=0.15$ . These parameters were determined in multiple preliminary simulations to be those that permit the EA to find the best solution. The inset figures represent the minimum (blue line) and maximum (red line) plasma glucose concentrations 2 h after a meal, and the gray area represents the optimal basal glucose concentrations for patients with diabetes. The maximum plasma glucose concentrations 2 h after a meal should have been <180 mg/dL, according to the ADA criteria, and we found that the EA was able to find an optimal solution for each individual, tailored to their particular characteristics.

**FIGURE 3:**
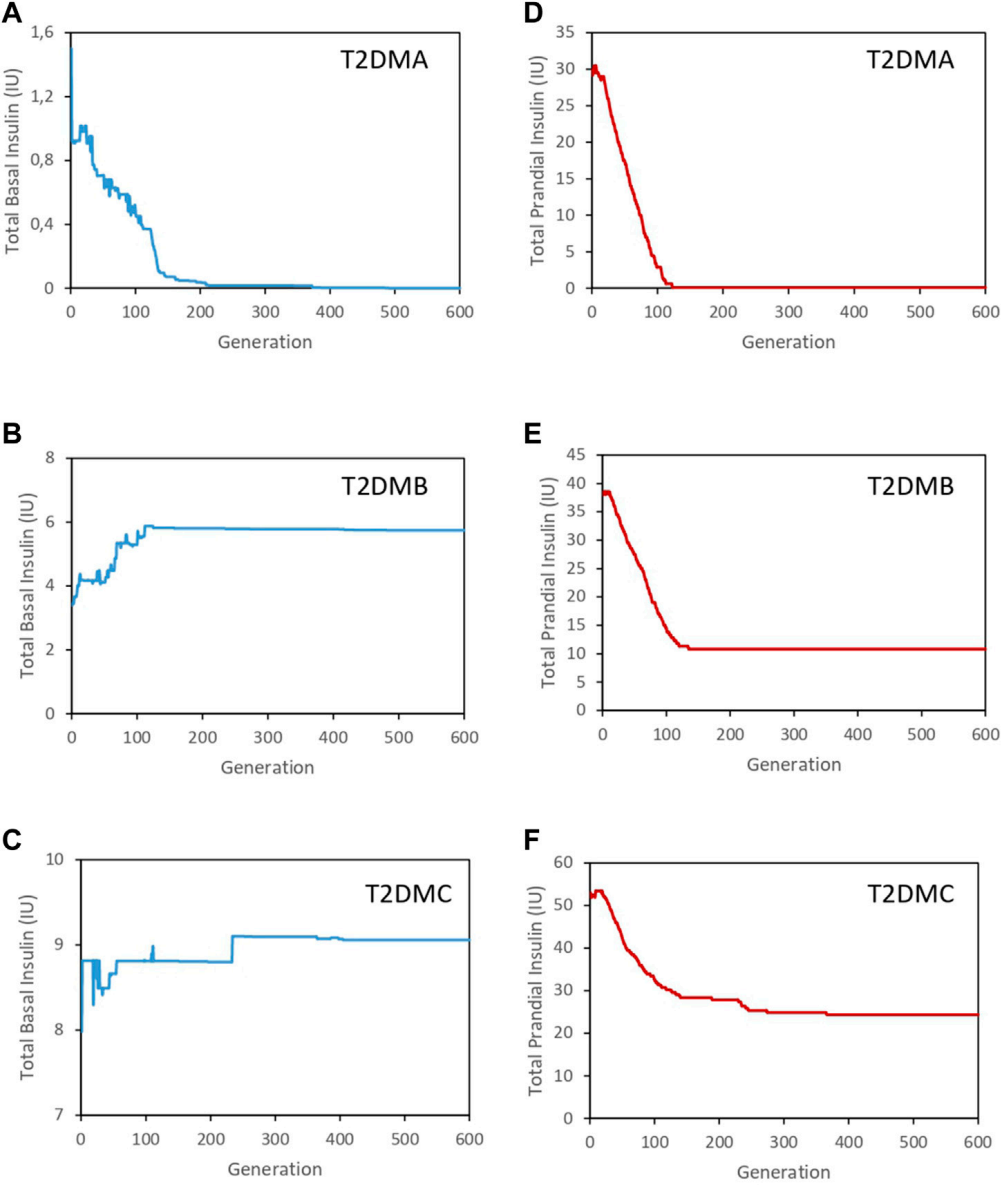
Evolution of the total insulin dose required for the three individuals analyzed. **(A).** Evolution of the basal insulin dose for T2DMA (blue line). **(B).** Evolution of the basal insulin dose for T2DMB (blue line). **(C)**. Evolution of the basal insulin dose for T2DMC (blue line). **(D)**. Evolution of the prandial insulin dose for T2DMA (red line). **(E).** Evolution of the prandial insulin dose for T2DMB (red line). **(F).** Evolution of the prandial insulin dose for T2DMC (red line).

The minimization of the fitness function was associated with that of the amount of insulin required to control glycemia, as shown in Figure 3. Figures 3A–C show the evolution of the basal insulin doses provided to ensure optimal glucose concentrations prior to a meal for the three individuals analyzed. In addition, Figures 3D–F show the evolution of the prandial insulin doses administered. Initially, the doses of prandial insulin were high in the three individuals analyzed. However, as the dietary intake patterns were modified by the EA (see Supplementary Figures S3, S4, and Supplementary Figures S5), superior regulation could be achieved alongside a reduction in the amount of insulin required. It is important to note that in the case of the individual T2DMA, the solution provided by the algorithm did not require the administration of exogenous insulin. The optimal distribution of food intake throughout the day was sufficient to achieve a good level of regulation of glycemia in this individual, whereas the individuals T2DMB and T2DMC required increasing doses of both basal and prandial insulin, according to the severity of T2DM exhibited by each individual.

Providing the EA with total freedom regarding the determination of intake patterns, with respect to both the timing and size of meals, permitted it to identify solutions that would not be easily implemented by a real patient. Therefore, to explore how the imposition of restrictions on the dietary intake patterns would affect the solutions identified by the EA, two new sets of simulations were performed.

**FIGURE 4:**
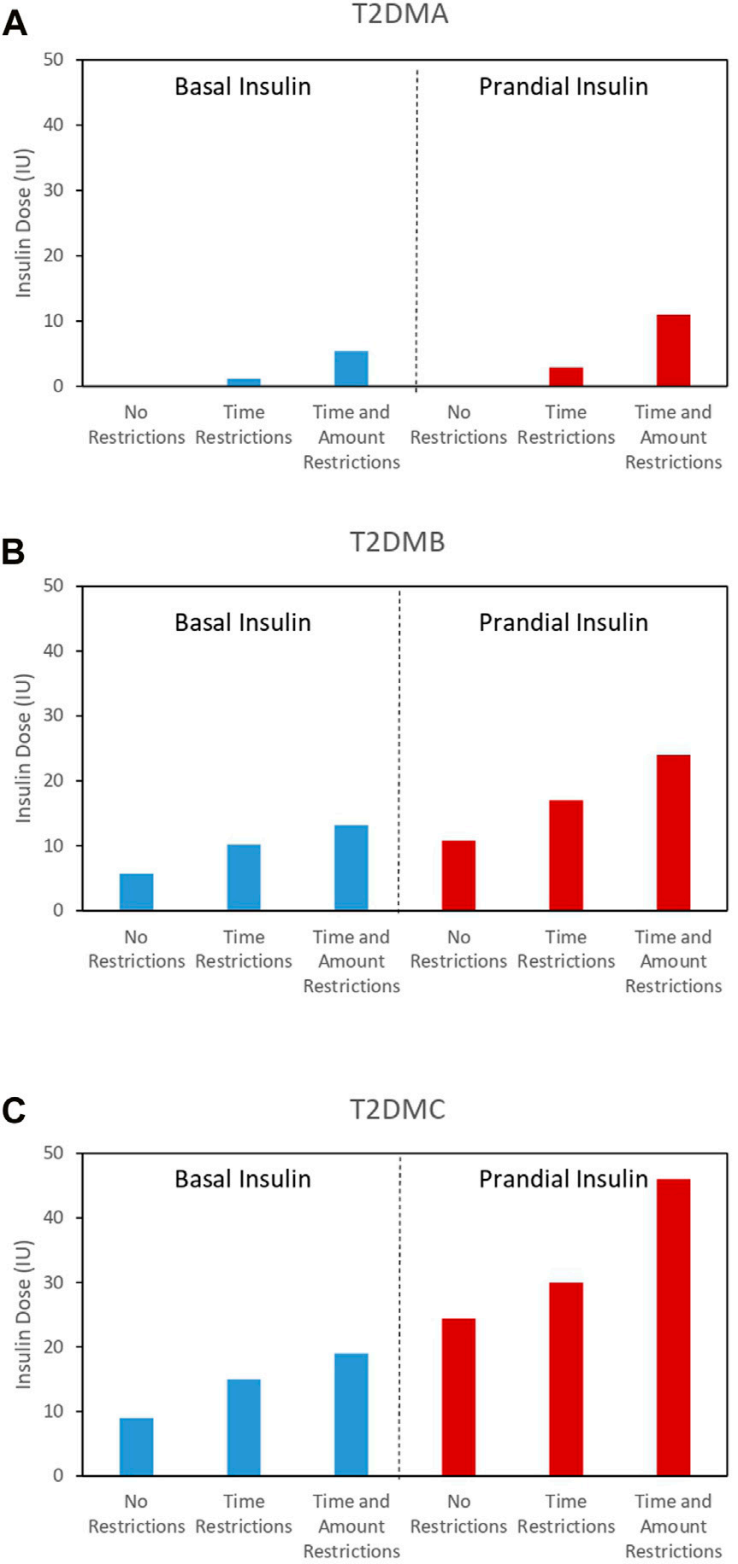
Minimum basal (blue bars) and prandial (red lines) insulin doses required for optimal glycemic control under scenarios with differing levels of restrictions for all the individuals analyzed. **(A)** T2DMA, **(B)** T2DMB, and **(C)** T2DMC.

Initially, a time-restricted scenario was analyzed, in which a uniform temporal distribution of meals, at 06:00, 10:00, 13:00, 17:00, and 20:00, was imposed. As in the previous round, we found that after 600 generations, the fitness function was stable (Supplementary Figure S6) and associated with good glycemic control, in terms of both the basal and postprandial glucose concentrations (insets in Supplementary Figure S6). Figure 4 shows the total doses of insulin required for glycemic control in the time-restricted scenario. In this set of simulations, the restrictions affecting the temporal distribution of meals did not increase the insulin doses required, indicating that a uniform temporal distribution of meals assists with glycemic control.

Subsequently, a more restrictive scenario was analyzed, in which both the timing and size of meals were fixed. Specifically, the timing of the five meals was fixed at 06:00, 9:00, 12:00, 16:00, and 19:00, as previously, and the amounts of glucose ingested were fixed at 35 g, 15 g, 70 g, 20 g, and 55 g, respectively. Despite the EA only being able to optimize the insulin dose under these conditions, the fitness function was again successfully minimized (Supplementary Figure S7) and remained stable after 600 generations, implying good glycemic control, with respect to both the basal and postprandial glucose concentrations (insets in Supplementary Figure S7). However, the solutions obtained by the EA involved increases in both the prandial and basal insulin doses required in the three individuals analyzed (Figure 4). Supplementary Figure S8, 9 shows the time evolution of glucose with optimized distribution of meals in the time restricted scenario and time and quantity restricted scenario respectively. Furthermore, Supplementary Figures S10, S11 show the evolution of the basal insulin doses (blue lines) and prandial insulin doses (red lines) corresponding to the time restricted scenario and the time and quantity restricted scenario respectively. It should be noted that the individual T2DMA required insulin administration to achieve optimal glucoregulation, whereas in the previous unrestricted scenario, the same level of regulation could be achieved without insulin.

In general, the results shown in Figure 4 indicate that the imposition of restrictions had a negative effect on glycemic control in all the individuals analyzed. This negative effect necessitated increases in the prandial and basal insulin doses.

**FIGURE 5:**
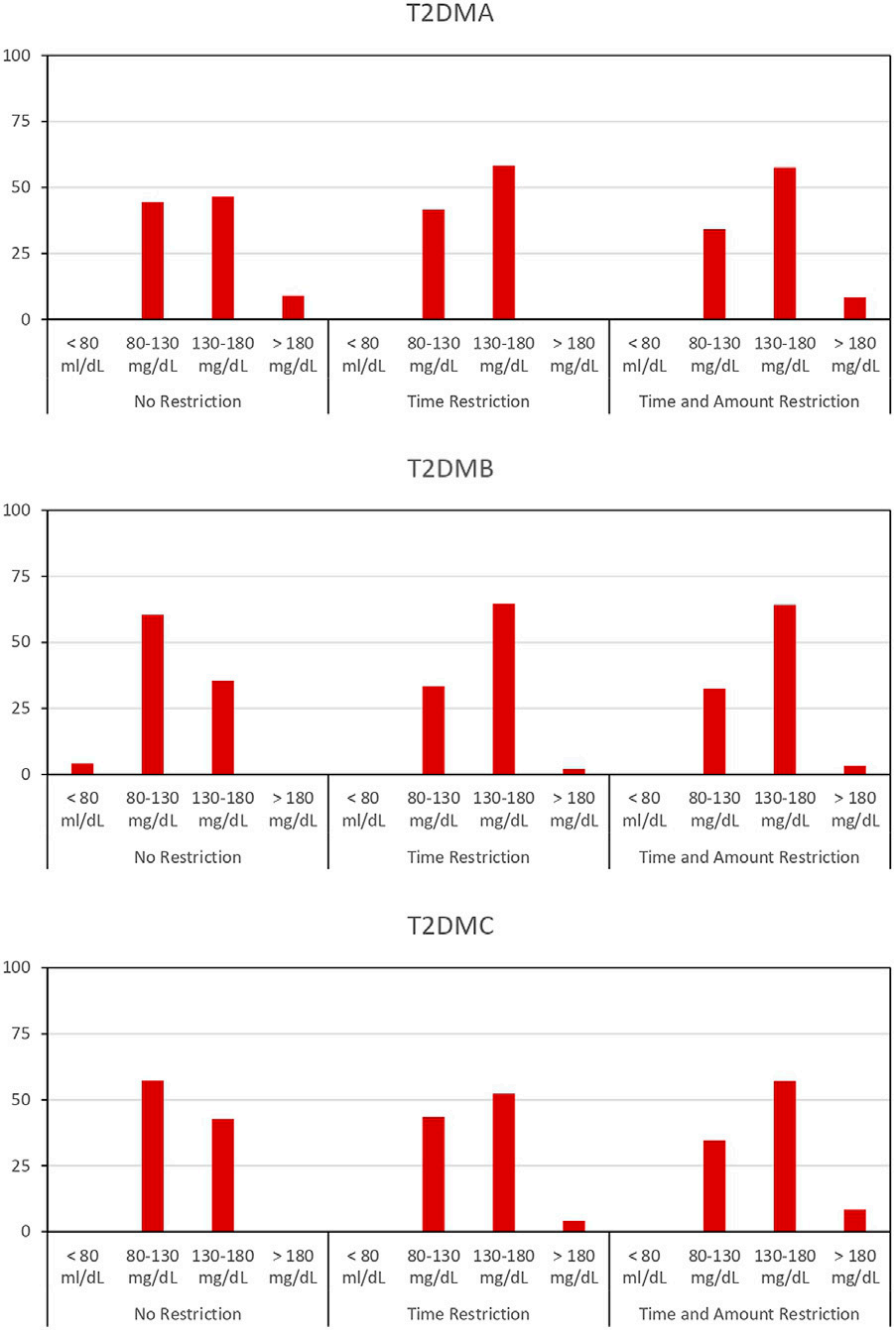
% of time at different glucose intervals for the subjects T2DMA, T2DMB and T2DMC: i) below 80 mg/dL, ii) 80–130 mg/dL, iii) 130–180 mg/dL and iv) above 180 mg/dL upon different restrictive scenarios.

Figure 5 provides a summary of the effects of EA optimization. This represents the % of daily time at different glucose intervals for the test subjects, namely, i) below 80 mg/dL, ii) 80–130 mg/dL, iii) 130–180 mg/dL and iv) above 180 mg/dL. The results show that most of the time, individuals take between 80 and 130 mg/dL and between 130 and 180 mg/dL. However, it should be mentioned that time intervals with glucose below 80 mg/dL, glucose levels are very close to 80 mg/dL and no episodes of hypoglycemia are observed. Similarly, when glucose exceeds 180 mg/dL, it remains very near this value and no episodes of hyperglycemia are observed.

## 4 Discussion

Recent advances in AI, in combination with more precise mathematical models of physiological systems, represent significant advances toward a personalized medicine that are applicable to many diseases. In this context, in the present study, we have evaluated the utility of EA for the optimization of glycemic control in patients with T2DM. This was achieved by combining an appropriate temporal distribution of meals with appropriate exogenous insulin doses. The objective of the EA was to minimize the insulin doses required to achieve appropriate glycemic control, and this reduction could be achieved by appropriately distributing meals throughout the day. Three types of individuals were analyzed, with various severities of diabetes.

In all the cases studied, the EA successfully established patterns of food intake and insulin doses, both basal and prandial, that optimized glycemic control according to the characteristics of the individuals considered.

Three scenarios were considered. In the first scenario, the EA was free to modify the timing and size of meals, as well as the insulin doses, to achieve glycemic control according to the ADA criteria. Subsequently, a more restrictive scenario was considered, in which the timing of meals was predetermined, such that the EA could modify only the size of each meal and the doses of insulin administered. Finally, we analyzed the most restrictive scenario, in which both the timing and size of meals were fixed, and the EA was only able to determine the insulin doses used.

We found that the prandial insulin dose required depended not only on the total amount of glucose ingested during the day but also on the temporal distribution of meals. However, it should be noted that the basal insulin requirement was not affected by meal distribution. Furthermore, for the individual T2DMA, it was possible to control glycemia by optimizing meal distribution alone, without the necessity for exogenous insulin administration, consistent with this individual being prediabetic. However, as the freedom to determine meal intake pattern was reduced, the requirement for prandial insulin increased.

The present findings highlight two important aspects of the control of T2DM: i) the importance of dietary management and ii) the great potential for the use of mathematical models of metabolic systems and EA in the treatment of diseases such as diabetes.

The applicability of results obtained in the optimization of glycaemia through an evolutionary strategy can be limited by the limitations inherent in the physiological model used, in our case the model based on the work of (Visentin et al. (2020). For example, the model used in this study did not consider the specific characteristics of the various types of insulin in use. In addition, it will be necessary to develop more refined models that consider other aspects such as the influences of types of carbohydrates other than glucose and other sources of energy, such as fatty acids or the effects of physical exercise. Finally, the roles of counter-regulatory hormones, such as glucagon, epinephrine, and growth hormone, have not been considered in the present model.

It should be noted that the fact of having carried out a first study only on three average subjects represents a limitation of the generality of the results obtained. Consequently, these results can only be considered as a preliminary proof of concept of the potential use of the evolutionary strategies for glycemic regulation.

However despite the limitations of the present study, the results suggest that the use of an evolutionary strategy can be useful in optimizing the regulation of glucose in type 2 patients through an optimization of the daily meal distribution and the doses of insulin applied. Future work should focus on the validation of the proposed evolutionary strategy, extending this study to other insulin models or other types of treatments, such as oral drugs. Moreover, these studies should analyze the performance of the evolutionary strategy considering a wide range of different patients, in order to perform a statistical analysis that support the preliminary findings presented in this work.

In summary, the combination of a mathematical model of metabolic systems that considers the characteristics of specific diabetic patients and EA may represent a significant advance toward personalized medicine for patients with diabetes.

## Data Availability

The raw data supporting the conclusion of this article will be made available by the authors, without undue reservation.
