# Supplementary material for: Evolutionary algorithm for the optimization of meal intake and insulin administration in patients with type 2 diabetes
Source: Front Physiol. 2023 Apr 6;14:1149698. doi: 10.3389/fphys.2023.1149698 (PMC10115945; doi:10.3389/fphys.2023.1149698)
Supplement: Supplementary file 1 [file DataSheet1.PDF]

EVOLUTIONARY ALGORITHM FOR MEAL AND INSULINE OPTIMIZATION IN DIABETES TYPE II

Eva Gonzalez-Flo[1], Elaheh Kheirabadi[1], Carlos Rodriguez-Caso[2,3], Javier Macía[1,*]

[1]Synthetic Biology for Biomedical Applications. Department of Medicine and Living Sciences, Universitat Pompeu Fabra, Barcelona, Spain

[2]Department of Molecular Biology and Biochemistry, Faculty of Sciences, University of Malaga, Andalucía Tech, E-29071 Malaga, Spain.

[3]IBIMA (Biomedical Research Institute of Malaga), E-29071 Malaga, Spain.

Correspondence: Javier Macia*

E-mail: javier.macia@upf.edu

PHYSIOLOGICAL MODEL

The physiological model used in the simulations of this study is an adaptation of the models developed by (Visentin, Cobelli and Dalla Man, 2020) and (Schiavon *et al.*, 2020). This model involves different subsystems described below

Glucose Subsystem

This subsystem describes the kinetics of glucose, differentiating plasma glucose and rapid equilibrium tissues, Gp, and glucose in slow equilibrium tissues, Gt.

The following equations describe the kinetics of glucose.

$$\dot{G_p}(t) = -k_1 G_p(t) + k_2 G_t(t) + EGP(t) + Ra(t) - U_{ii} - E(t)$$

$$\dot{G_t}(t) = k_1 G_p(t) - k_2 G_t(t) - U_{id}(t) \tag{S.1}$$

$$G(t) = \frac{G_p(t)}{V_G}$$

Here:

- V_G is the distribution of glucose volume

- G is the concentration of glucose in the blood plasma
- G_p is glucose in plasma and fast-balance tissues
- G_t is plasma glucose and slow-balancing tissues
- EGP is endogenous glucose production
- E is renal excretion
- Ra is the rate of plasma glucose appearance
- U_{ii} is the use of insulin-independent glucose
- U_{id} is the use of insulin-dependent glucose
- k_1 and k_2 are model parameters.

Glucose rate of appearance

The description of the intestinal glucose absorption process differs between the amount of glucose present in the stomach, Q_{sto}, and in the gut, Q_{gut}. It is also necessary to differentiate between the glucose that is in the stomach in solid state and that which is in triturated state. Intestinal glucose absorption is modeled by a three-compartment model:

$$\begin{aligned} \dot{Q}_{sto1}(t) &= -k_{gri}Q_{sto1}(t) + d(t) \\ \dot{Q}_{sto2}(t) &= -k_{empt}(Q_{sto}(t))Q_{sto2}(t) + k_{gri}Q_{sto1}(t) \\ \dot{Q}_{gut}(t) &= -k_{abs}Q_{gut}(t) + k_{empt}(Q_{sto}(t))Q_{sto2}(t) \\ Q_{sto}(t) &= Q_{sto1}(t) + Q_{sto2}(t) \\ Ra(t) &= \frac{f \cdot k_{abs}Q_{gut}(t)}{BW} \end{aligned} \tag{S.2}$$

with

$$k_{empt}(Q(t)) = k_{min} + \frac{k_{max} - k_{min}}{2}\{\tanh[\alpha'(Q_{sto}(t) - dD(t))] - \tanh[\beta'(Q_{sto}(t) - dD(t))]\} \tag{S.3}$$

$$\alpha' = \frac{5}{2D(t)(1-b)}$$

$$\beta' = \frac{5}{2D(t)d} \tag{S.4}$$

$$D(t) = \int_{t_i}^{t_f} d(t)dt$$

Here:

- Q_{sto} is the amount of glucose in the stomach (solid phase, Q_{sto1}, and triturated phase, Q_{sto2})
- Q_{gut} is the mass of glucose in the intestine
- Ra is the rate of plasma glucose appearance
- K_{gri} is the grinding rate
- K_{max} is the maximum gastric emptying rate
- K_{min} is the minumum gastric emptying rate
- k_{abs} is the constant rate of intestinal absorption
- f is the fraction of intestinal absorption that actually appears in the plasma
- d is the amount of glucose ingested
- BW is the body weight
- k_{empt} is the gastric emptying rate constant
- t_i is the starting time of the meal
- t_f is the ending time of the meal

Glucose Renal Excretion

Renal excretion represents the flow of glucose that is eliminated by the kidney, when

glycaemia exceeds a certain threshold, according to:

$$E(t) = \begin{cases} k_{e1} \cdot \left[G_p(t) - k_{e2}\right] & if\ G_p(t) > k_{e2} \\ 0 & if\ G_p(t) \leq k_{e2} \end{cases} \tag{S.5}$$

Here:

- k_{e1} is the renal glomerular filtration rate
- k_{e2} is the maximum glucose threshold
- G_p is glucose in plasma and tissues of rapid equilibrium
- E is renal excretion.

Insulin Subsystem

Insulin flow *s(t)*, which comes from the subcutaneous compartments, is incorporated into the blood flow and degraded in the liver and periphery, according to:

$$\dot{I_p}\ (t) = -(m_2 + m_4 + m_5)I_p(t) + m_1 I_l(t) + m_6 I_{ev}(\mathrm{t}) + S(t)$$

$$\dot{I_l}\ (t) = -(m_1 + m_3(t)) \cdot I_l(t) + m_2 I_p(t) + ISR(t)/BW \tag{S.6}$$

$$\dot{I_{ev}} = -m_6 I_{ev}(t) + m_5 I_p(t)$$

$$I(t) = I_p(t)/V_l$$

with

$$HE(t) = -\mathrm{a}_{\mathrm{G}} \cdot G(t) + \mathrm{a}_{0\mathrm{G}} \tag{S.7}$$

and

$$m_3(t) = \frac{HE(t) \cdot m_1}{1 - HE(t)} \tag{S.8}$$

Here:

- V_l is the volume of insulin distribution

- I_p is the mass of insulin in plasma
- I_l is the insulin mass in the liver
- I_{ev} is the insulin mass in the extravascular space
- m_1, m_2, m_3 and m_4 are model parameters
- I is the plasma insulin concentration

Subsystem of subcutaneous insulin

In diabetic patients, insulin is usually given by subcutaneous injection. The system is described by a model of two compartments, S_1 and S_2 (pmol/kg), which represent, respectively, multimeric and monomeric insulin in the subcutaneous tissue.

The model considers two types of insulin. On the one hand basal insulin is supplied by an insulin pump with an infusion ratio $u_b(t)$. On the other hand, prandial insulin $u_p(t)$ is injected instantly *at* t_p.

$$\begin{aligned} \dot{S_1}(t) &= -(k_{a1} + k_d)S_1(t) + u_b(t\text{-}\tau) + u_p(t_p)\,\delta(t_p+\tau) \\ \dot{S_2}(t) &= k_d S_1(t) - k_{a2}S_2(t) \\ S(t) &= k_{a1}S_1(t) + k_{a2}S_2(t) \end{aligned} \quad \text{(S.9)}$$

Here

$$\delta(t_p + \tau) = \begin{cases} 1 & if\ t = t_p + \tau \\ 0 & otherwise \end{cases}$$

- t_p is the time of prandial insulin injection
- S_1 is the polymeric insulin in the subcutaneous tissue
- S_2 is monomeric insulin in subcutaneous tissue
- k_d is the degradation constant
- k_{a1} and k_{a2} are the absorption constants of polymeric and monomeric insulin, respectively.

- τ is the delay in the appearance of insulin in the first compartment (insulin in a non-monomeric state)

Endogenous glucose production

Endogenous glucose production (EGP) takes place in the liver, where glycogen is transformed into glucose. EGP is inhibited by high levels of glucose and insulin.

$$EGP(t) = k_{p1} - k_{p2}G_p(t) - k_{p3}I_d(t) - k_{p4}I_l(t) \quad \text{(S.10)}$$

Here

- k_{p2} is the parameter that measures the effectiveness of glucose in the liver
- k_{p3} is the parameter that regulates the amplitude of insulin action in the liver
- k_{p3} is the parameter related with delayed insulin
- G_p (mg/kg) is glucose in plasma and tissues of rapid balance
- I_d (pmol/l) is the delayed insulin signal according to:

$$\begin{aligned} \dot{I_1}(t) &= k_i I(t) - k_i I_1(t) \\ \dot{I_d}(t) &= k_i I_1(t) - k_i I_d(t) \end{aligned} \quad \text{(S.11)}$$

where:

- k_i is the parameter that counts the delay between the insulin signal and its action

Glucose Utilization

Glucose can be consumed in two different ways: i) independently of insulin, U_{ii}, which represents the absorption of glucose by the brain and erythrocytes and is considered constant; and ii) insulin-dependent, U_{id}, which depends nonlinearly on glucose in slow balance tissues.

Hence:

$$U(t) = U_{ii} + U_{id}(t) \tag{S.12}$$

$$U_{ii}=F_{cns}$$

where:

$$U_{id}(t) = V_m(X(t))\frac{(1 + r_1 \cdot risk) \cdot G_t(t)}{K_{m_0} + G_t(t)} \tag{S.13}$$

$$risk = \begin{cases} 0 & if\ G \geq G_b \\ 10 \cdot [f(G)]^{\,2} & if\ G_{th} \leq G \leq G_b \\ 10 \cdot [f(G_{th})]^{\,2} & if\ G \leq G_{th} \end{cases} \tag{S.14}$$

$$f(g) = [\log(G)]^{r_2} - [\log(G_b)]^{r_2} \tag{S.15}$$

Here V_m is a function that depends linearly on interstitial insulin X,

$$V_m(X(t)) = V_{m0} + V_{mx}X(t) \tag{S.16}$$

which depends on insulin levels:

$$\dot{X}(t) = -p_{2U}X(t) + p_{2U}(I(t) - I_b) \tag{S.17}$$

Here

- K_m, V_m and V_{mx} are parameters defining the functional dependence between $U_{id}(t)$ and $G_t(t)$
- I_b is the basal insulin level
- p2U is the rate of action of insulin in peripheral glucose

Subsystem of Insulin Secretion

The secretion rates of total $ISR(t)$, static $ISR_s(t)$, dynamic $ISR_d(t)$ and basal insulin $ISR_b(t)$ (and C-peptide) from the beta-cells are described by:

$$ISR(t)= ISR_s(t) + ISR_d(t) + ISR_b(t) \tag{S.18}$$

Here:

$$\dot{ISR_s}(t) = -\alpha[ISR_s(t) - V_c \cdot \beta \cdot (G(t) - h)] \qquad \text{(S.19)}$$

$$ISR_d(t) = \begin{cases} V_c \cdot K \cdot \dot{G}(t) & if\ \dot{G}(t) \geq 0 \\ 0 & if\ \dot{G}(t) < 0 \end{cases} \qquad \text{(S.20)}$$

$$ISR_b(t) = CP_b \cdot k_{01} \cdot V_c \qquad \text{(S.21)}$$

- α is the delay between glucose signal and insulin secretion
- β is the pancreatic responsivity to glucose
- K is the β-cell responsivity to the glucose rate of change
- *h* is the threshold level of glucose above which the β-cells initiate to produce new insulin

REFERENCES

Schiavon, M. *et al.* (2020) ‘Modeling Subcutaneous Absorption of Long-Acting Insulin Glargine in Type 1 Diabetes’, *IEEE Transactions on Biomedical Engineering*, 67(2), pp. 624–631. doi:10.1109/TBME.2019.2919250.

Visentin, R., Cobelli, C. and Dalla Man, C. (2020) ‘The Padova Type 2 Diabetes Simulator from Triple-Tracer Single-Meal Studies: In Silico Trials Also Possible in Rare but Not-So-Rare Individuals’, *Diabetes Technology and Therapeutics*, 22(12), pp. 892–903. doi:10.1089/dia.2020.0110.

Table S1. Parameters used in the physiological model for individuals with type 2 diabetes

	**Parameter**	**Value**
Glucose Subsystem	V_G (dL/Kg)	1.00
	k_1 (min^{-1})	0.066
	k_2 (min^{-1})	0.043
Insulin Subsystem	a_G (dL/mg)	0.005
	Vl (L/Kg)	0.041
	m_1 (min^{-1})	0.314
	m_2 (min^{-1})	0.268
	m_4 (min^{-1})	0.443
	m_5 (min^{-1})	0.260
	m_6 (min^{-1})	0.017
Glucose Rate of Appearance	k_{abs} (min^{-1})	0.0542
	k_{gri} (min^{-1})	0.0462
	k_{max} (min^{-1})	0.0462
	k_{min} (min^{-1})	0.0076
	G_{th} (mg/dL)	60
	b	0.73
	d	0.1
	f	0.9
Glucose Renal Excretion	k_{e1} (min^{-1})	0.0005
	k_{e2} (mg/Kg)	339

	**Parameter**	**Value**
Glucose Utilization	$Fcns$ (mg/kg/min)	1
	V_{m0} (mg/kg/min)	4.65
	V_{mx} (mg/kg/min per pmol/L)	Table 1
	K_{m0} (mg/Kg)	0.034
	p_{2U} (min^{-1})	0.058
Insulin Secretion	K (10^{-9})	Table 1
	α	0,034
	β (10^{-9} min^{-1})	Table 1
	h (mg/dL)	98.7
	Vc (L)	4.18
	k_{01} (min^{-1})	0.053
	r_1	1.794
	r_2	1.026
Subcutaneous Insulin	τ (min)	7.6
	k_{a1} (min^{-1})	0.0034
	k_{a2} (min^{-1})	0.014
	k_d (min^{-1})	0.028
	CP_b (nmol/L)	0.9
Endogenous Glucose Production	k_i (min^{-1})	0.0075
	kp_2 (min^{-1})	0.0008
	kp_3 (mg/kg/min per pmol/L)	Table 1
	kp_4 (mg/kg/min per pmol/kg)	0.0484

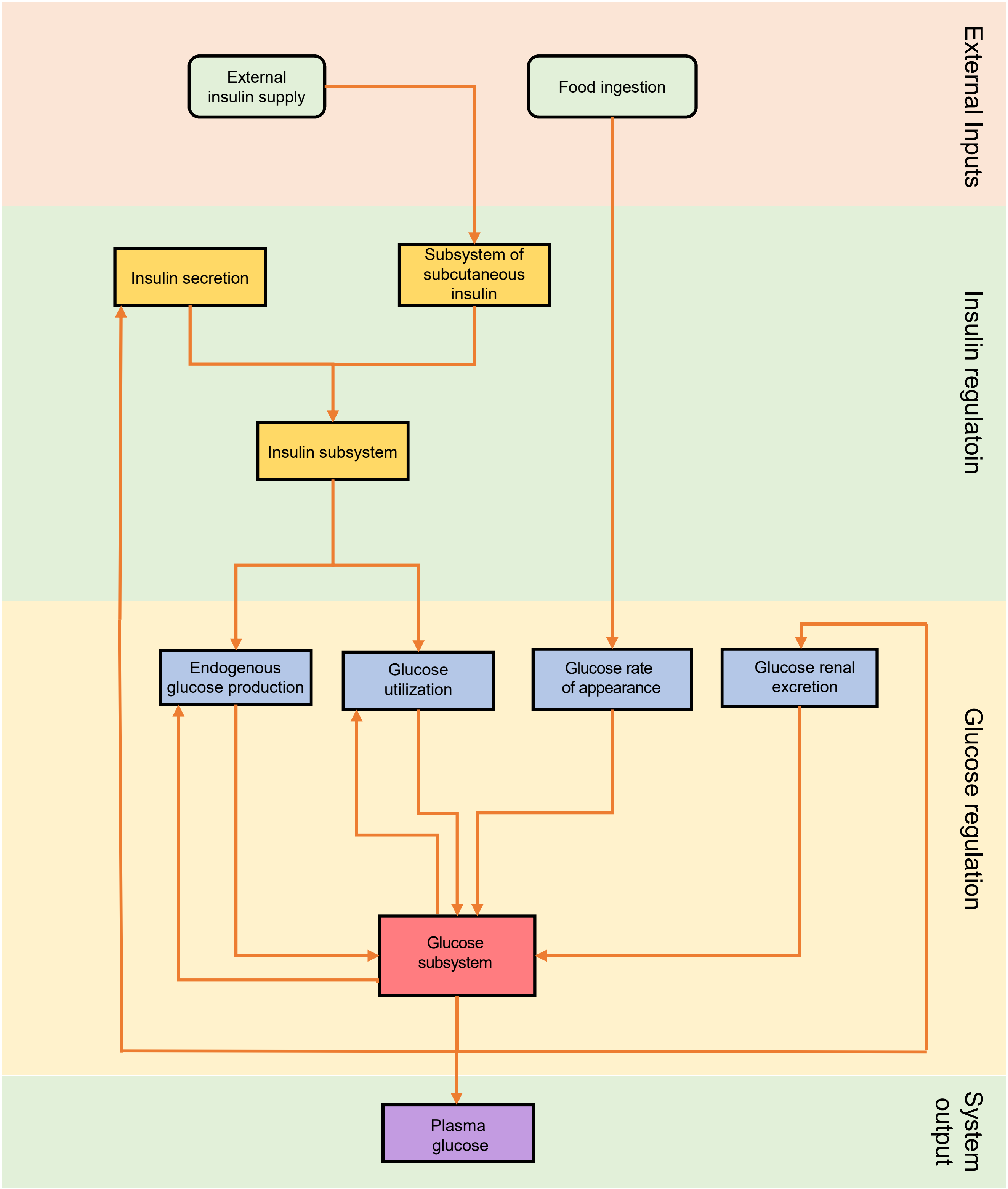

Figure S1. Schematic representation of the physiological model subsystems and their interactions

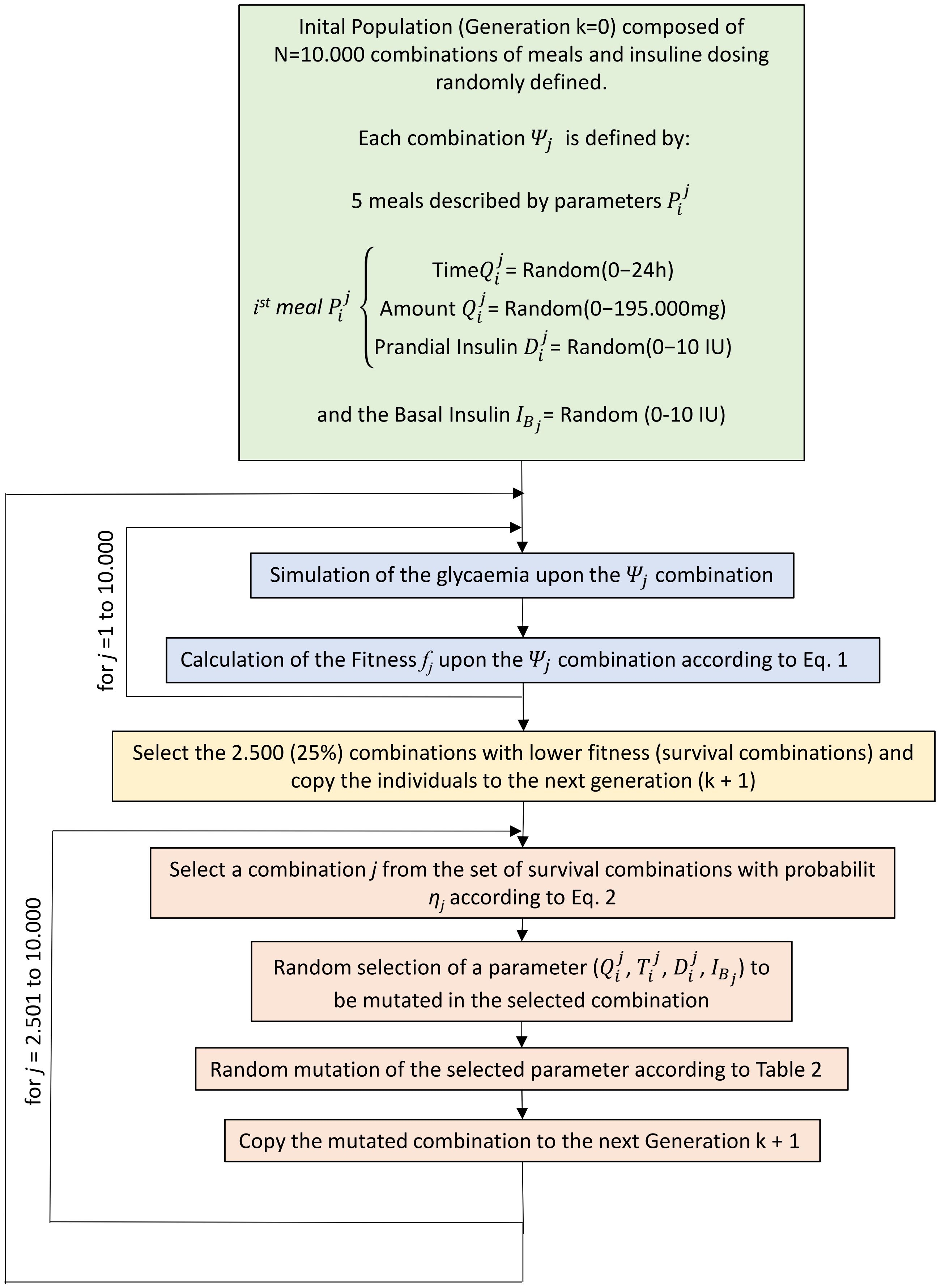

Figure S2. Flow chart of the evolutionary algorithm

T2DMA

Generation 0

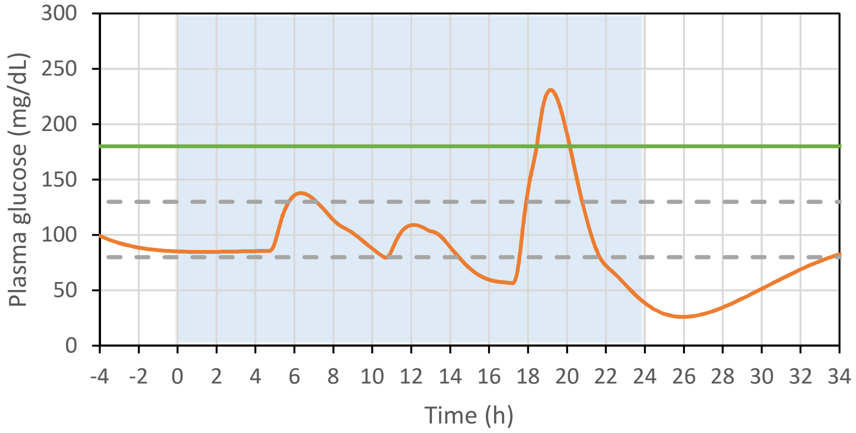

T (h)	A(g)
4,9	39,8
11,1	30,7
13,5	0,4
17,9	88,2
19,2	35,9

Generation 100

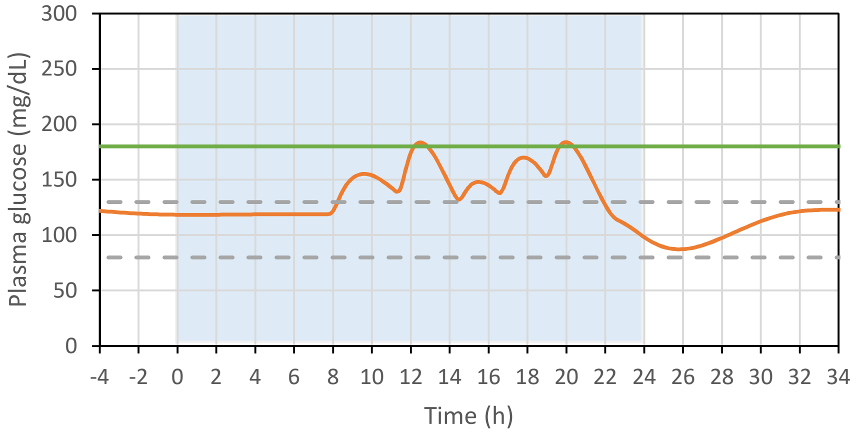

T (h)	A(g)
8,0	25,9
11,8	58,1
14,9	28,2
17,1	35,8
19,8	47,0

Generation 600

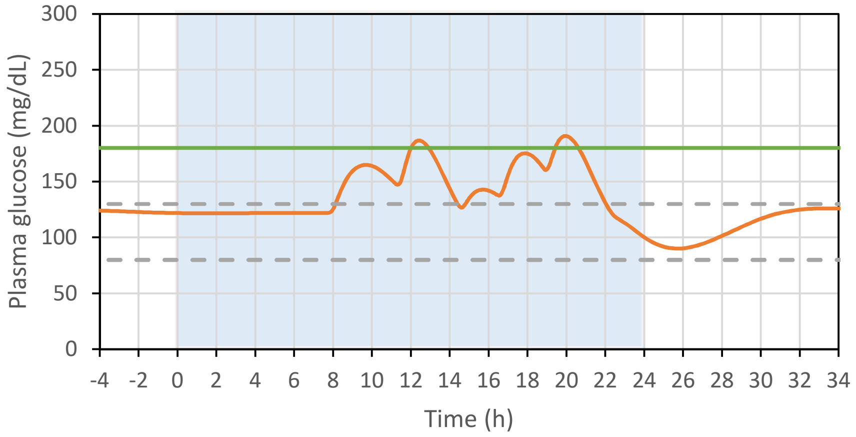

T (h)	A(g)
8,1	28,0
11,8	57,9
15,0	26,1
17,1	37,2
19,8	45,7

Figure S3. Evolution of the meal intake pattern performed by the evolutionary algorithm for the individual T2DMA. The blue area represents the day of study. In the tables, the timing (T) and amount (A) of glucose consumed are shown. The gray dashed lines represent normoglycemia interval and the green line is the limit of hyperglycemia 2h after meal consumption (180 mg/dL), according to the American Diabetes Association criteria.

T2DMB

Generation 0

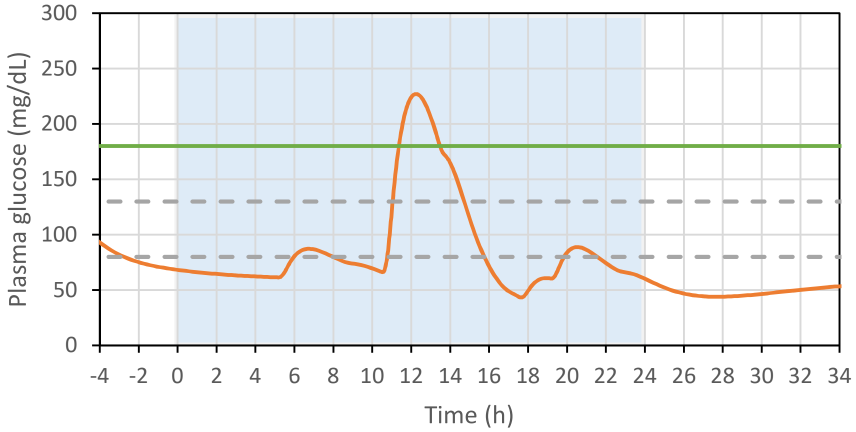

T (h)	A(g)
5,4	21,3
11,0	114,9
14,1	12,0
18,2	22,7
20,1	24,2

Generation 100

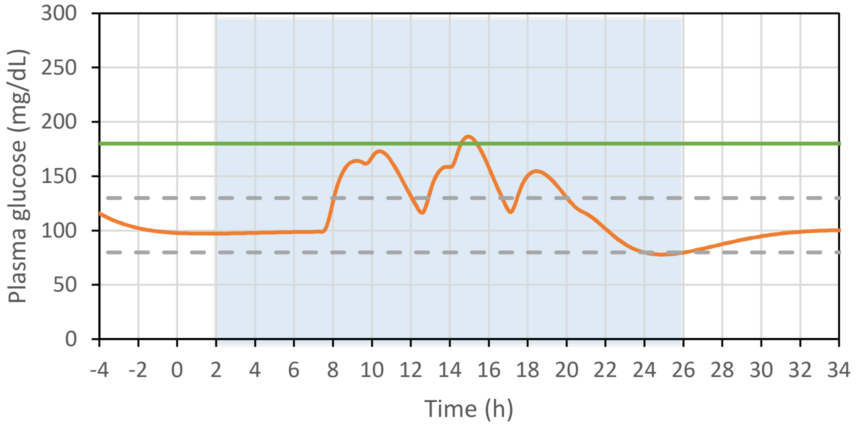

T (h)	A(g)
7,5	45,6
9,9	33,2
12,8	38,3
14,5	35,9
17,7	42,1

Generation 600

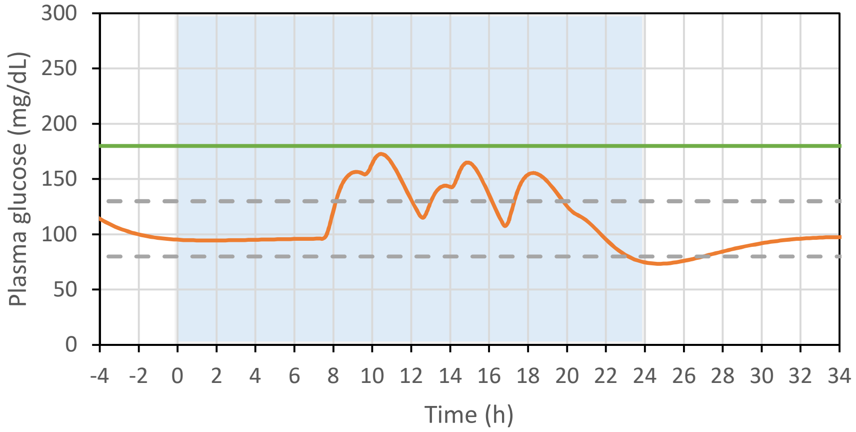

T (h)	A(g)
7,5	42,6
9,9	31,4
12,8	38,1
14,5	35,7
17,4	47,2

Figure S4. Evolution of the meal intake pattern performed by the evolutionary algorithm for the individual T2DMB. The blue area represents the day of study. In the tables, the timing (T) and amount (A) of glucose consumed are shown. The gray dashed lines represent normoglycemia interval and the green line is the limit of hyperglycemia 2 h after meal consumption (180 mg/dL), according to the American Diabetes Association criteria.

T2DMC

Generation 0

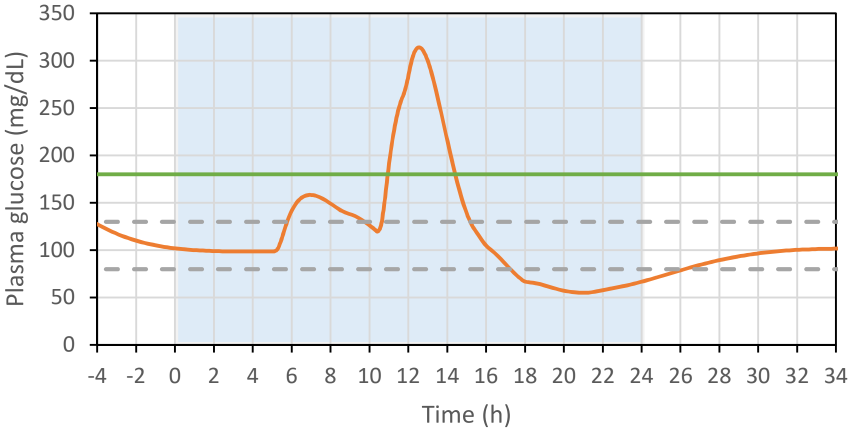

T (h)	A(g)
5,3	40,2
10,9	113,4
12,4	25,3
16,4	10,2
18,8	6,0

Generation 100

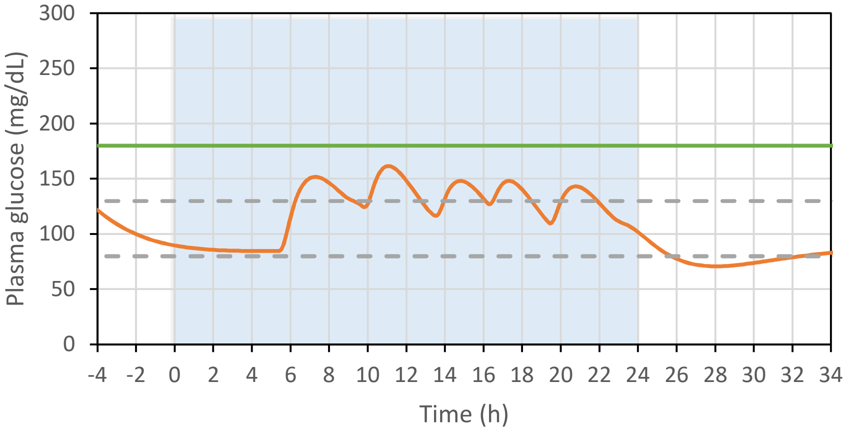

T (h)	A(g)
5,4	43,8
10,1	45,9
13,8	36,9
16,7	33,5
20,1	34,9

Generation 600

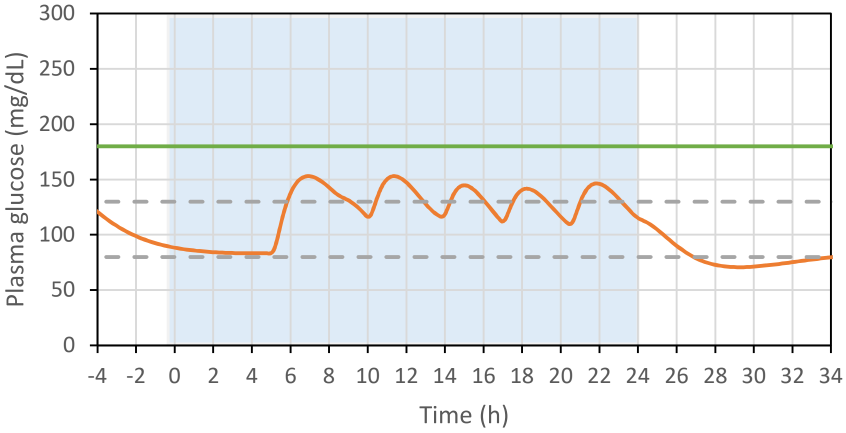

T (h)	A(g)
5,0	43,2
10,2	43,1
14,1	37,4
17,3	34,0
21,1	37,3

Figure S5. Evolution of the meal intake pattern performed by the evolutionary algorithm for the individual T2DMC. The blue area represents the day of study. In the tables, the timing (T) and amount (A) of glucose consumed are shown. The gray dashed lines represent normoglycemia interval and the green line is the limit of hyperglycemia 2 h after meal consumption (180 mg/dL), according to the American Diabetes Association criteria.

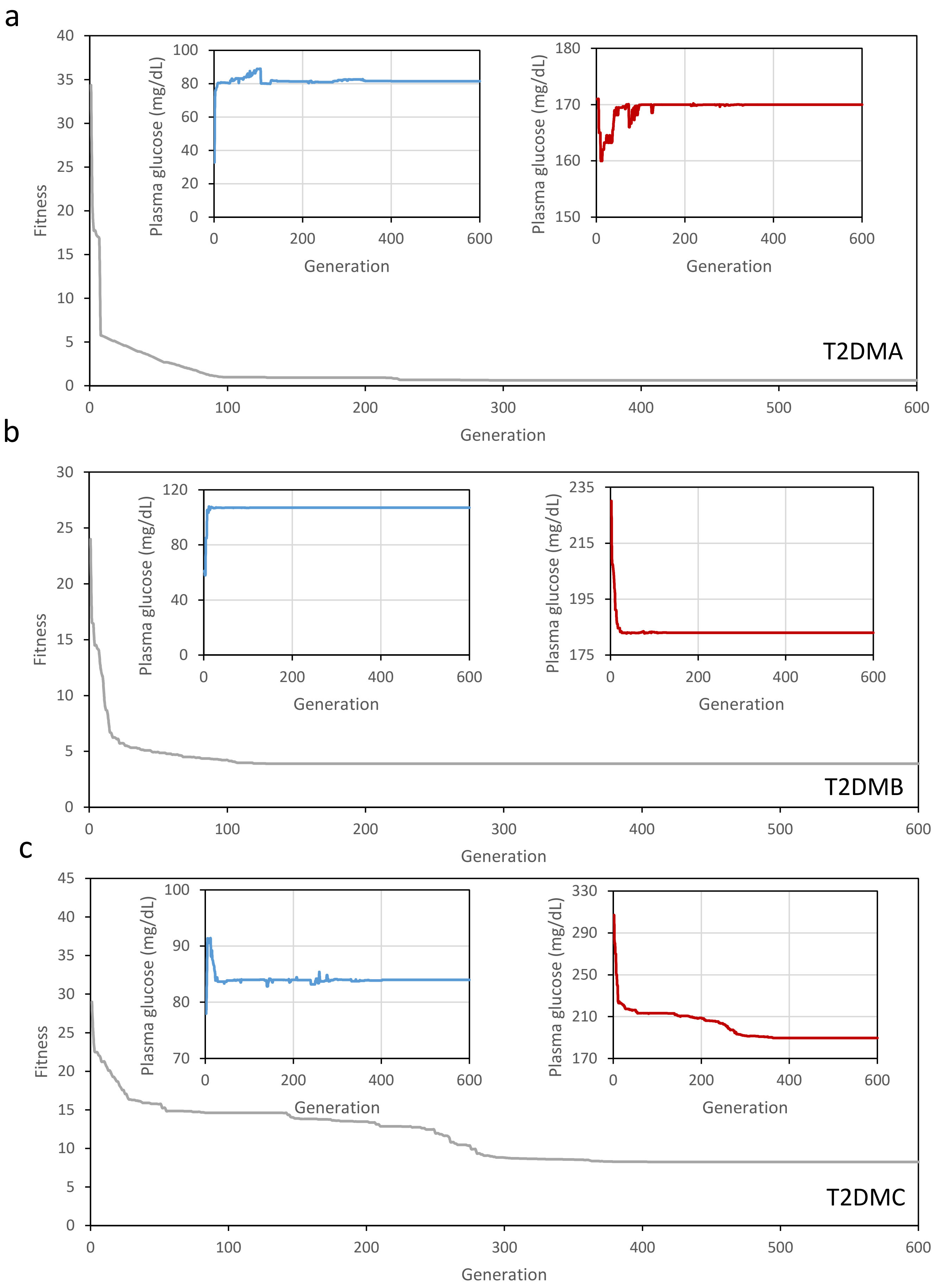

Figure S6. Evolution of the fitness function over 600 generations for the 3 individuals, (**a**) T2DMA, (**b**) T2DMB, and (**c**) T2DMC, under the time-restricted scenario. Simulations were performed using the parameters $\mu_1 = 0.35$, $\mu_2 = 0.35$, and $\mu_1 = 0.15$. Inset figures represent the minimum (blue line) and maximum (red line) plasma glucose concentrations 2 h following glucose intake. The gray area represents the optimal basal glucose concentrations for patients with diabetes, according to the American Diabetes Association criteria.

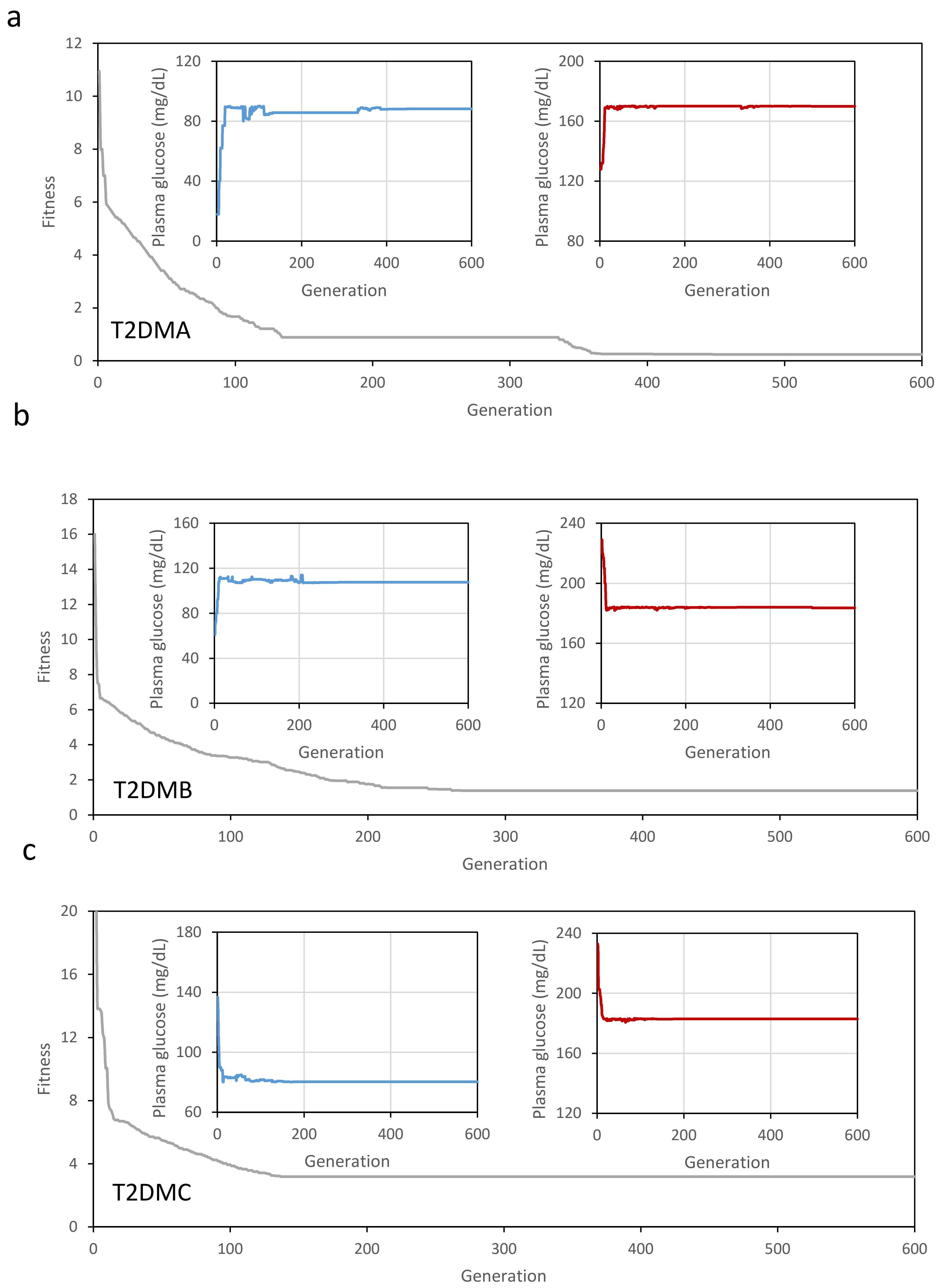

Figure S7. Evolution of the fitness function over 600 generations for the 3 individuals, (**a**) T2DMA, (**b**) T2DMB, and (**c**) T2DMC, under the time and glucose quantity-restricted scenario. Simulations were performed using the parameters $\mu_1 = 0.35$, $\mu_2 = 0.35$, and $\mu_1 = 0.15$. Inset figures represent the minimum (blue line) and maximum (red line) plasma glucose concentrations 2 h following glucose intake. The gray area represents the optimal basal glucose concentrations for patients with diabetes, according to the American Diabetes Association criteria.

Time Restriction

T2DMA

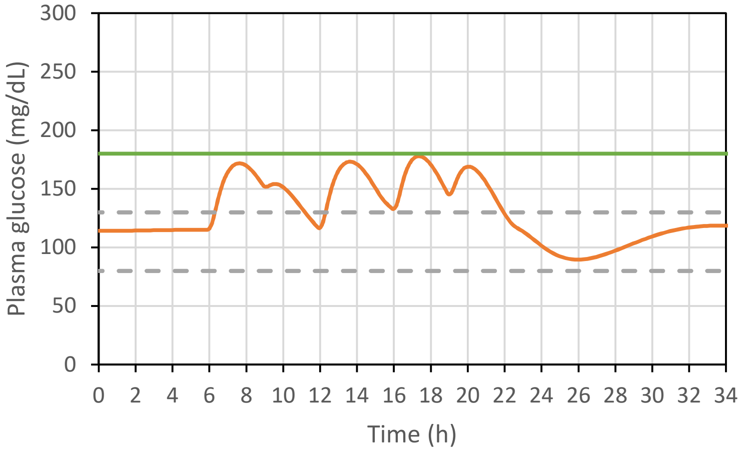

T (h)	A(g)
6	42,9
9	14,9
12	48,0
16	51,2
19	38,0

T2DMB

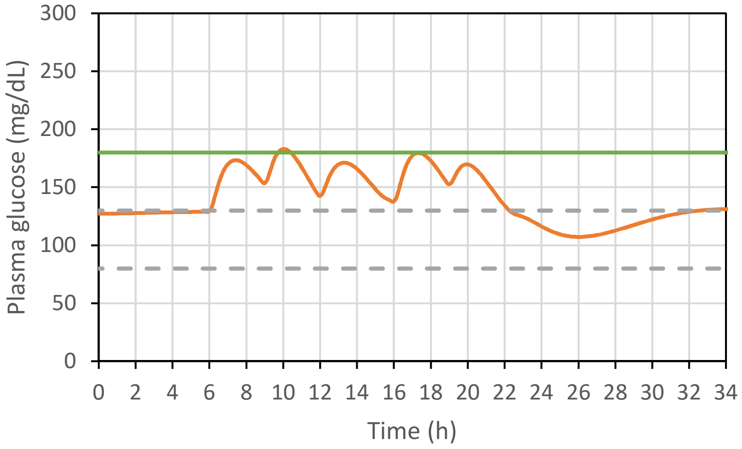

T (h)	A(g)
6	43,3
9	33,9
12	47,0
16	31,1
19	38,0

T2DMC

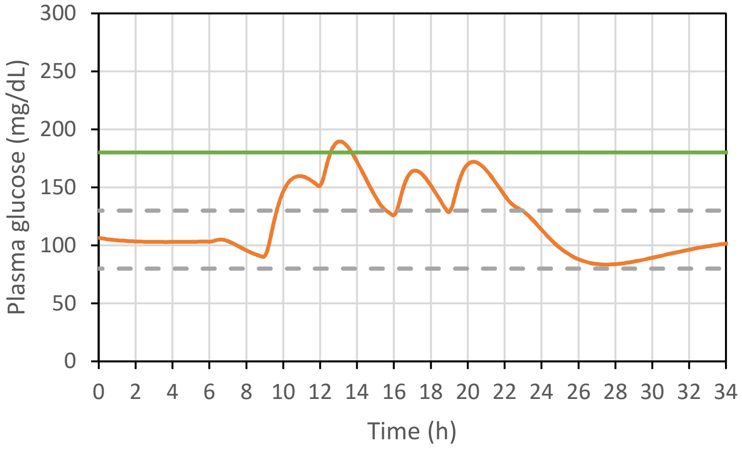

T (h)	A(g)
6	7,3
9	44,8
12	47,7
16	50,5
19	44,7

Figure S8. Time course of glucose with optimized distribution of meals in time restricted scenario. The gray dashed lines represent normoglycemia interval and the green line is the limit of hyperglycemia 2h after meal consumption (180 mg/dL), according to the American Diabetes Association criteria.

Time and Amount Restriction

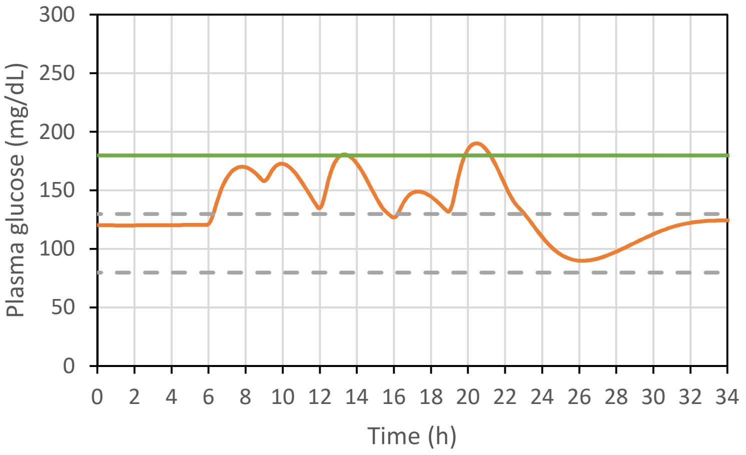

T (h)	A(g)
6	35
9	15
12	70
16	20
19	55

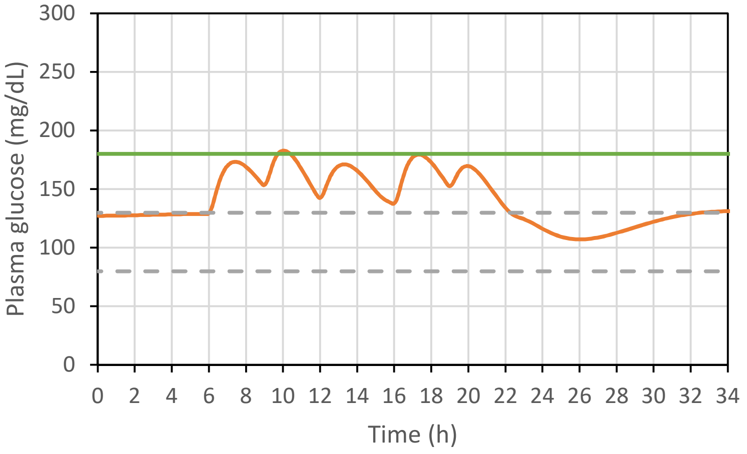

T (h)	A(g)
6	35
9	15
12	70
16	20
19	55

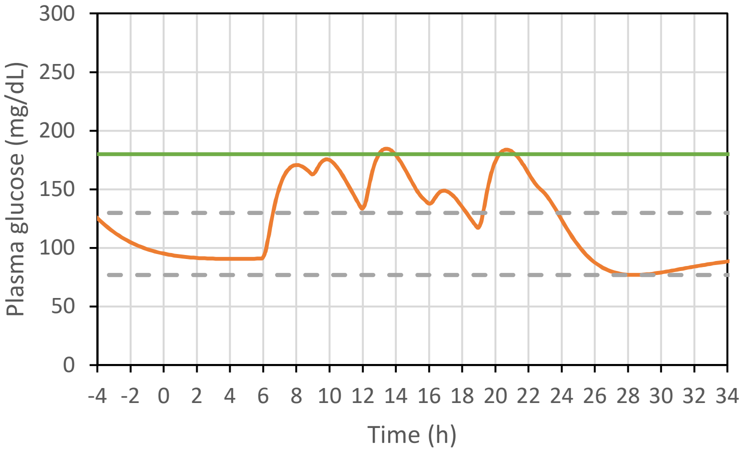

T (h)	A(g)
6	35
9	15
12	70
16	20
19	55

Figure S9. Time course of glucose with optimized distribution of meals in time and quantity restricted scenario. The gray dashed lines represent normoglycemia interval and the green line is the limit of hyperglycemia 2h after meal consumption (180 mg/dL), according to the American Diabetes Association criteria.

Time Restriction

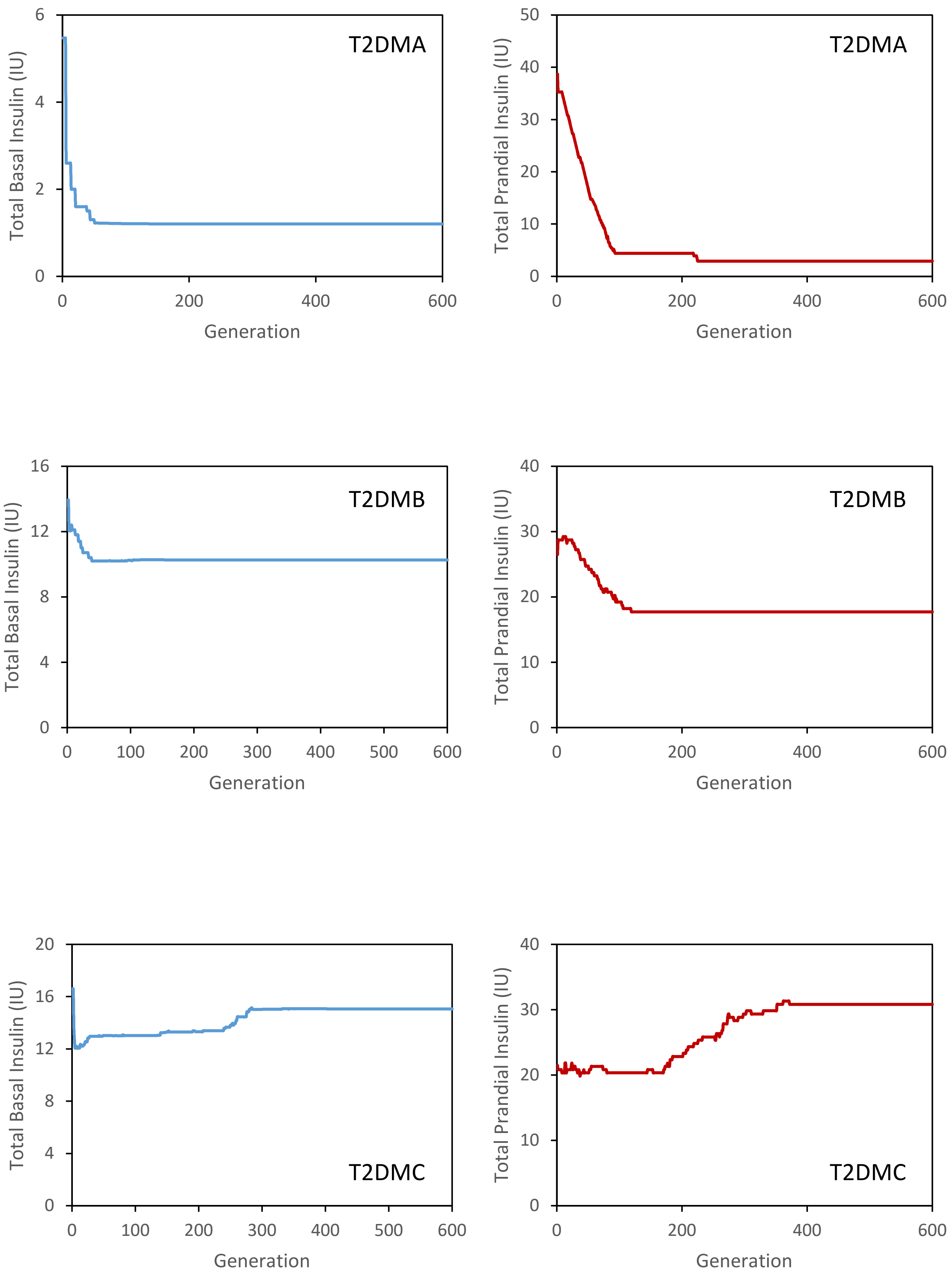

Figure S10. Evolution of the total insulin dose required for the three individuals analyzed in a time restricted scenario. Red line represents the evolution of the prandial insulin and the blue line the evolution of the basal insulin dose.

Time and Amount Restriction

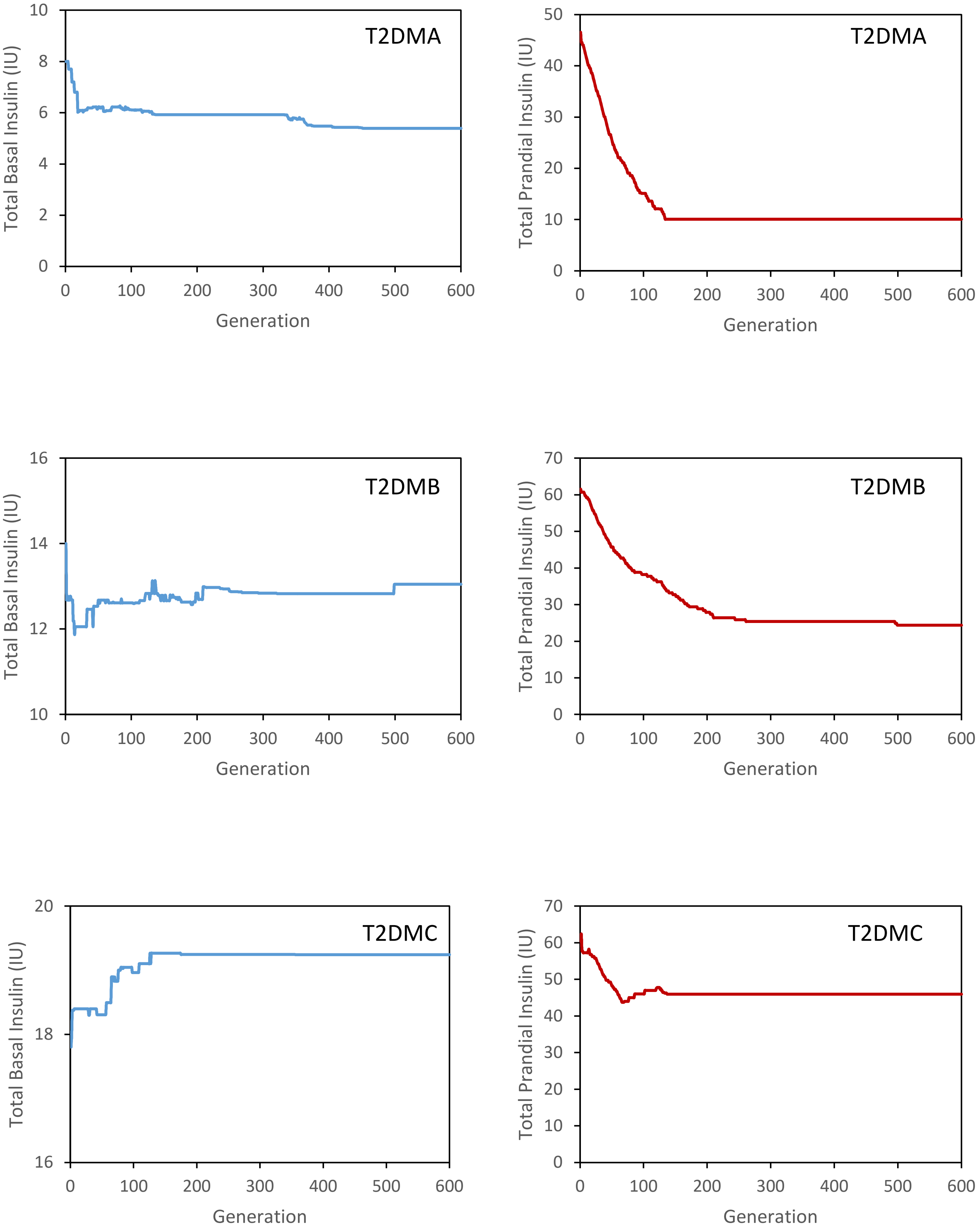

Figure S11. Evolution of the total insulin dose required for the three individuals analyzed in a time and amount restricted scenario. Red line represents the evolution of the prandial insulin and the blue line the evolution of the basal insulin dose.
